# Enterobius vermicularis in appendectomy specimens; Clinicopathological assessment: Cross sectional study

**DOI:** 10.1016/j.amsu.2020.10.057

**Published:** 2020-10-28

**Authors:** Abdulkarim Hasan, Khalid Nafie, Samar El-Sayed, Mohamed Nasr, Ayman Abdulmohaymen, Mohamed Baheeg, Osama Abbadi

**Affiliations:** a Department of Pathology, Faculty of Medicine, Al-Azhar University, Cairo, Egypt; b Laboratory & Blood Bank Department, Prince Mishari Bin Saud Hospital, Baljurashi, Saudi Arabia; c Department of Parasitology, Faculty of Medicine for Girls, Al-Azhar University, Cairo, Egypt; d Department of Histology, Faculty of Medicine, Al-Azhar University, Cairo, Egypt; e Department of Surgery, Faculty of Medicine, Al-Azhar University, Cairo, Egypt; f Department of Biochemistry, Faculty of Medicine, Omdurman Islamic University, Sudan; g Department of Surgical Oncology Faculty of Medicine, Al-Azhar University, Cairo, Egypt

**Keywords:** Appendix, Histopathology, Parasitic infestation

## Abstract

**Background:**

This study identifies the incidence of appendiceal Enterobius vermicularis (E.v) infestation in all the patients undergoing appendectomy and evaluates the relationship between E. v infestation of the appendix and the acute appendicitis.

**Method:**

ology: All the routinely examined appendectomy specimens received in the pathology laboratory of a referral hospital over a three year period of time were reviewed for the existence of E. v. These cases were evaluated for clinico-laboratory characterization.

**Results:**

Out of 1150 appendectomies for clinical acute appendicitis picture, 31 (2.7%) cases revealed E. v infestation. The age ranged from 6 to 42 years old but more than 80% of the E. v infected cases were children. Twenty four cases (77.4%) did not show any other appendiceal pathology, six cases showed lymphoid hyperplasia and only one case showed concomitant histological acute inflammatory process.

**Conclusion:**

E. v infestation is an incidental finding during histopathology examination of appendectomy specimens for patients with clinical diagnosis of acute appendicitis, however there is no relation between the existence of E. v and occurrence of acute appendicitis which is the main indication for appendectomy, so further studies are recommended to reach out earlier diagnosis to eliminate the unnecessary surgical intervention. Also surgeons should consider E. v as a differential diagnosis when removing a normal looking appendix to take the necessary precautions for minimizing any chance of contamination and sending all the normal looking appendectomy specimens for histopathology examination.

## Highlights

• E. vermicularis was seen in 2.7% of all the resected appendices.
• More than 96% of cases were not associated with any acute inflammatory cells.
• Surgical intervention can be avoidable.

## 1 Introduction

Enterobius vermicularis (E.v.) infestation, also known as Pinworm infection, is a common parasitic disease affecting about 200 million people around the world^1^. It commonly affects children [1,2]. The cecum is the major site for E. v. to live and the gravid female usually migrates at night to lay up to several thousands of eggs [3]. It can reach the appendix and might cause serious morbidity results from appendicitis; however parasitic infestation of the appendix itself is rare [4].

The role of this worm in the etiology of acute appendicitis is controversial; some authors concluded that the presence of pinworms in the appendix may clinically mimic acute appendicitis but most likely incidental rather than being an etiology of acute appendicitis [4].

The clinical mimicking of acute appendicitis may result in a person undergoing an unnecessary negative surgery (appendectomy), also predicting the presence of E. v. is important to ensure complete treatment with proper anti-helminthic drugs [5], and to prevent dissemination of the infection at the time of surgery [6].

In this study, we aimed to identify the incidence of E. v. infestation in all the patients undergoing appendectomy in three hospitals performing the pathology examination in our laboratory department over three consecutive years and also to study the relative clinico-laboratory characterization of the patients suffering from pinworm disease of the appendix.

## 2 Materials and methods

A total of 1150 reports of appendectomy specimens that were diagnosed in our referral pathology laboratory department in a three-year period (between April 2017 and April 2020) were retrospectively scanned to identify the cases with E. v. infection. Hematoxylin-eosin (H&E) stained slides for 31 appendectomies with E. v. infestation were reexamined by the pathology author for the presence of acute inflammation, eosinophils, lymphoid hyperplasia and parasitic infestation. Pathology request forms usually contain relative clinical data [26] , so we retrieved the patient's forms and medical files to record other relevant predictive factors for E. v. including: age, gender, complete blood count (CBC) and gross features. We report the results of this study in accordance with STROCCS reporting statements [7]. Statistical Analysis was performed using the Microsoft Excel 2007 software. Test of significance was performed by *t*-test with two samples-assuming equal variances. For correlation assessment, Pearson's test was performed and according to Evans, 1996 [8] the strength of correlation was detailed as follows: 0.00–0.19: very weak; 0.20–0.39: “weak; 0.40–0.59: moderate; 0.60–0.79: strong; and between 0.80 and 1.0: very strong correlation.

## 3 Results

**Fig. 1:**
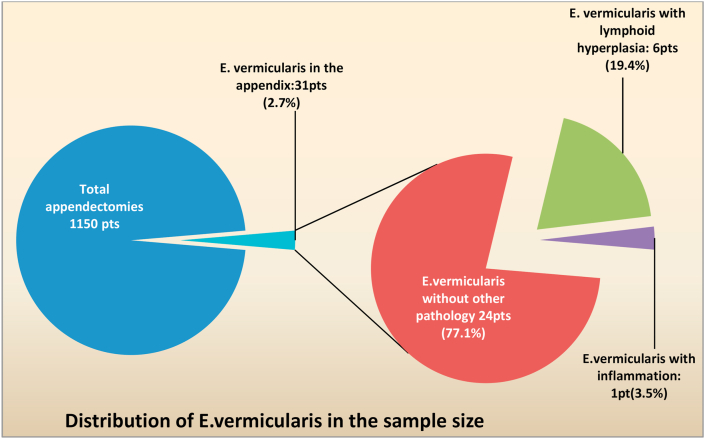
A pie of pie chart highlighting the fraction of patients with appendectomies, from a total of 1150, whom had E. vermicularis in their appendices. The side Pie chart shows the percents of three diagnostic categories of these E. vermicularis patients.

**Fig. 2:**
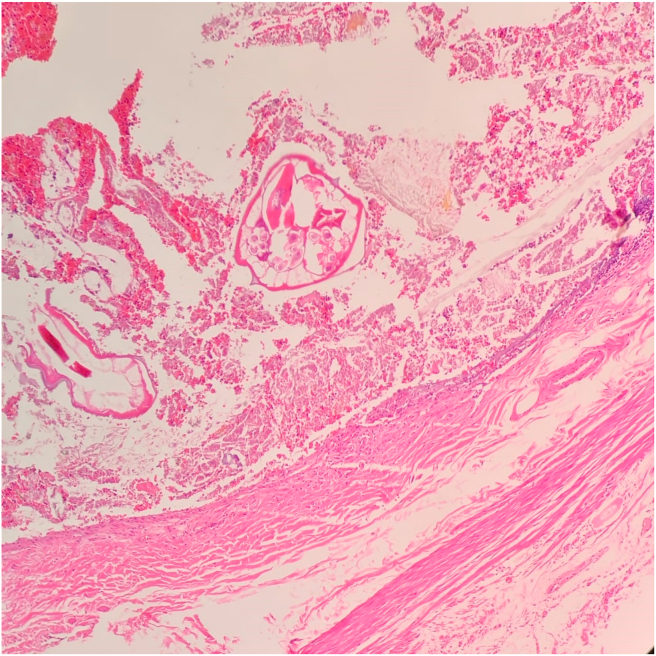
A histopathology picture of a case of appendiceal section showing luminal E. vermicularis with no evidence of acute inflammatory process (H&E stained, 200x).

**Fig. 3:**
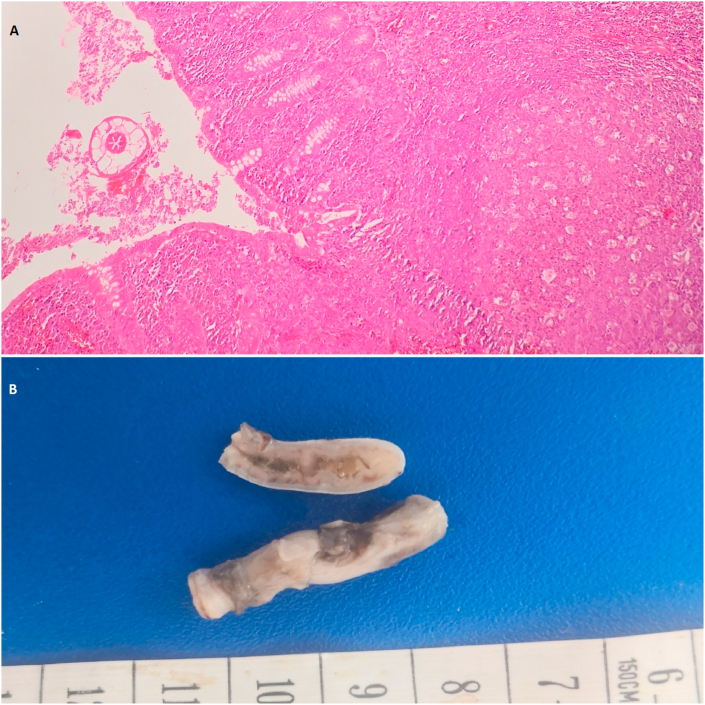
A) A histopathology picture of a case of appendiceal section showing luminal E. vermicularis with lymphoid hyperplasia (H&E stained, 100x), B) A gross picture of non-inflammed appendix with E. v.

Out of 1150 specimens with a clinical diagnosis of acute appendicitis, 31 (2.7%) contained E. v. at histopathology examination (Fig. 1), When the studied 31 E. v cases evaluated in terms of histopathology diagnoses, most of them; 24 cases (77.1%) revealed Enterobius vermicularis without any other pathology (Fig. 2), six cases (19.4%) were associated with lymphoid hyperplasia (L.H) (Fig. 3) and only one case (3.5%) showed acute inflammatory process, 13 out of 31 (42%) were male, and 18 (58%) were female. Patient's age ranged from 6 to 42 years with mean age 15.5 ± 7.2 years. The appendicular length ranged from 35 mm up to 100 mm with mean length 61.6 ± 16.7 and the appendicular diameter ranges from 5 mm up to 7 mm with mean diameter 5.8 ± 0.75 (See Table 1).

**Table 1:** Summary of the descriptive results and *P*-values of significance with regard to the Sample population. A comparison in values between males and females were performed with testing of the significance of difference via *t*-test. *P*-value is considered significant at P < 0.05.

Parameter	Frequency	Mean Age	Mean Hemoglobin (g/dL)	Mean TWBCS (In 10^12^/L)	Mean Blood Eosinophils %	Length of the appendix by mm	Diameter of the appendix by mm
Males	13 (41.9%)	12.69 ± 5.15	12.69 ± 1.33	9.63 ± 3.99 × 10^12^/L	3.37 ± 3.9%	61.92 ± 14.94	5.75 ± 0.45
Females	18 (58.1%)	17.27 ± 8.04	12.96 ± 1.62	7.92 ± 3.21 × 10^12^/L	4.54 ± 4.37%	61.38 ± 18.38	5.83 ± 0.92
P value	–	0.08	0.6	0.2	0.45	0.93	0.77
Comments							

Analysis of CBC for the all studied cases showed; hemoglobin ranged from 8 up to 15 g/dL with the overall mean being 12.8 ± 1.5 g/dL; male mean was12.69 ± 1.33 g/dL and female mean was12.96 ± 1.62 g/dL; as seen in Table 1.

Total White blood cells (TWBCs) ranged from 3.6 × 10^12^/L up to 17 × 10^12^/L, with the male mean being 9.63 ± 3.99 × 10^12^/L and female mean 7.92 ± 3.21 × 10^12^/L. Eosinophilic percent ranged from 0.3% to 16.5% with a mean eosinophil percent of 3.37 ± 3.9% for males and 4.54 ± 4.37% for the females. All the differences in values between males and females were insignificant, since all *P*-values were in the range between 0.08 and 0.93 (See Table 1).

The correlation between the ages and genders of the patients to the other variables of the study: Appendix Length, Appendix diameter, Blood eosinophils percent, and the given histopathology diagnosis were either weak or very weak, and the lengths of the patients’ appendices particularly were negatively correlated to their ages and genders. Correlation between TWBCs and Blood eosinophils % and histopathology diagnosis also was performed; the eosinophils count was negatively correlated to the TWBCs count, and the histopathology diagnosis is very weakly correlated to the TWBCs count (Revise Table 2).

**Table 2:** Pearson correlations between Ages and genders of the participants and the following variables: Appendix length, appendix diameter, Blood eosinphils percent, and the final histopathology diagnosis. TWBCs also was correlated to the eosinophils % and the diagnosis.

Correlates	Appendix Length	Appendix diameter	Blood eosinophils %	Histopathology diagnosis
Patient's Age	−0.0666579	−0.1158740	0.0463557	−0.1148367
Patient's Sex	−0.015994	0.0429188	0.1412095	−0.21256643
TWBCs	–	–	−0.243173	0.1066423

## 4 Discussion

Parasites infests more than half of the humans on earth [4,9], most of these are in the intestinal tract [10]. It is in an endemic disease in Saudi Arabia as well as the developing countries especially in the rural areas [11].

Of all the parasites infesting human body, E. v. is the most common to be found in the gut, and also in appendices after resection [12]. Enterobius causing appendiceal pathology is known in medical literature for more than hundred years [13]. Based on the results of this research, the incidence of E. v. inhabiting a surgically removed appendix is 2.7%, which equals to 31 cases from the 1150 included specimen. The percent is high when compared to Yabanoğlu et al. study of 2014 where there were only 18 adult patients out of 1159 with parasitic infestation and 15 (1.29%) of those were E. v [4]. The current study, however, gave lesser prevalence than the 2015 Fleming et al. study which had been carried out in pediatric appendiceal samples; in a total of 182 samples, 14 (7.1%) were positive for Enterobius vermicularis [2] and this probably because E. v. is more common in children [1,2]. But the same percent recorded also by Ramezani and Dehghani in Iran who had reported E. v in 2.9% of surgically removed appendices [14].

A study in an age group of three to forty years gave a result of 0.74% of E. v. in histology examination, and this study of 2019 recruited 3222 appendectomy specimens [15]. Although the studies of correlation between acute appendicitis and the presence of E. v. inside the appendix usually yields one figure percentages, sometimes decimals, but the range is reportedly as wide as 0.2–41.8% around the globe [16]. No doubt the sanitation, geography, age, and country economics play role in these percents variations.

Of 31 E. v. found in the appendix specimens (portion of 1150 specimens) one only showed definite signs of acute inflammation in histopathology, which implies that inflammatory E. v. infestation of the appendix is as rare as less than one in thousand; the inflammation is attributed to secondary causes such as the parasite occupying the narrow appendiceal lumen, or the presence of other hidden pathology: fecolith, bacteria or other foreign body [17]. The only one case showing concomitant suppurative inflammation and E. v was seen obstructed with fecolith that is probably the etiology of the acute inflammation rather than the parasite. From the above, it is fair to speculate that E. v. would not cause true inflammation of the appendix. The conversation about whether E. v. elicits appendicitis was thoroughly studied, and researchers are suggesting it gives clinical mimicry to appendicitis but not typical inflammation [18,19]. Such remark is important since the treatment of E. v. is with drugs, not surgery [5,6].

The finding of females having higher prevalence of E. v. (58.1% of cases) comes in agreement with E. v. being more common in girls [5]. Eosinophils percent was within the normal range for both females and males (5% or less) [20]. Although eosinophils elevation is highly suggestive of parasitic infection, it is not a specific finding [21]. Eosinophilic cell infiltrate may be associated with neutrophilic infiltration in the gastrointestinal tract [22]. Lymphoid hyperplasia (L.H) was noticed in 6 of the 31 E. v. positive appendices which represents 19.4% of the cases; see Fig. 1. This is put in comparison with Pehlivanoğlu et al. study of 2019 where all the appendices with E. v. had accompanying LH [15]. Presence of parasite in appendiceal lumen can cause several pathologic conditions including LH leading to appendicitis-mimicking clinical symptoms [12,18].

There is a surgical debate for performing laparoscopic appendicectomy on a macroscopically normal appendix which found at time of surgery in clinically symptomatic patients and no other alternate pathology [6]. Some agreement is found within the literature revealing that this is justifiable decision [23]. There is also evidence that up to 50% of histologically normal appendices, showed expression of several inflammatory mediators such as PGE2, iNOS, COX2 and MHC class II expression [24]. The majority of surgical operations of the appendix are undertaken laparoscopically [25]. Surgeons during laparoscopic appendectomy procedure need to be aware of the possibility of parasitic infestation and simple techniques can minimize the contamination risk [6].

For the patients whose appendectomies were found to contain E. v, appendectomy alone is not the adequate treatment. Because surgery cannot abolish the cause but result a condition only, so patients must be advised anti-helminthic medications after surgery [4].

The limitation of this study includes the limited studied place as a single referral hospital was studied, also lack of comparison between the Intestinal E. v infestation and the initiation of acute appendicitis process.

## 5 Conclusion

E. v infection is an incidental finding in appendectomy specimens and may give similar clinical features of acute appendicitis but the high ratio of negative appendectomies containing E. v worms supports the hypothesis of no relation between the pinworm disease and acute appendicitis and both of them can give acute abdomen picture. Further studies are recommended to reach out earlier diagnosis of gastrointestinal parasitic to eliminate the unnecessary surgical intervention also surgeons should consider E. v as a differential diagnosis when removing a normal looking appendix to take the necessary precautions for minimizing any chance of contamination and to send the normal-looking specimen for histopathology examination.

## Ethical approval

Local ethical approval was obtained.

## Funding

This study did not receive any funding from governmental or private organizations.

## Author contribution

Study concept or design – AH, KH, SE.

Data collection – AH, KN, AA, MN.

Data interpretation – AH, OA.

Literature review – AH, SE, MB.

Data analysis – OA, KN.

Drafting of the paper – AH, KN, OA.

Editing of the paper – AH, KN, SE, MB, AA, MN.

## Registration of research studies

ClinicalTrials.gov Identifier: NCT04576273

## Guarantor

Dr. Abdulkarim Hasan

## Declaration of competing interest

No any conflict of interest in this study

## Availability of data and materials

The data that support the findings of this study are available from the corresponding author upon reasonable request.
